# Supplementary material for: Active site specificity profiling datasets of matrix metalloproteinases (MMPs) 1, 2, 3, 7, 8, 9, 12, 13 and 14
Source: Data Brief. 2016 Feb 22;7:299–310. doi: 10.1016/j.dib.2016.02.036 (PMC4777984; doi:10.1016/j.dib.2016.02.036)
Supplement: Supplementary file 10 — Supplementary material [file mmc10.zip › WebPICS_hMMP3_T_1%/P2.html]

 

PICS results


|  |  |
| --- | --- |
| **P2\_F**  9 in 199 sites   4.5 %    effects > 10 perc. pnts.  (vice-versa in brackets)  P1\_P: 24.3 (12.2)   P3prime\_H: 19.2 (28.8) |  |
| **P2\_G**  25 in 199 sites   12.6 %    effects > 10 perc. pnts.  (vice-versa in brackets)  P3\_V: 12.9 (14.7) |  |
| **P2\_L**  21 in 199 sites   10.6 %    effects > 10 perc. pnts.  (vice-versa in brackets)  P3\_H: 11.3 (39.4) |  |
| **P2\_M**  7 in 199 sites   3.5 %    effects > 10 perc. pnts.  (vice-versa in brackets)  P1\_F: 38.9 (34.0)   P2prime\_H: 11.3 (13.2)   P2prime\_M: 39.4 (39.4)   P3prime\_G: 56.3 (13.2) |  |
| **P2\_Q**  15 in 199 sites   7.5 %    effects > 10 perc. pnts.  (vice-versa in brackets)  P1\_S: 12.0 (11.3)   P1prime\_M: 10.3 (25.8)   P2prime\_I: 15.6 (10.7)   P3prime\_C: 11.8 (59.2) |  |
| **P2\_S**  14 in 199 sites   7.0 %    effects > 10 perc. pnts.  (vice-versa in brackets)  P1\_S: 27.7 (24.2)   P1prime\_W: 11.3 (26.3)   P3prime\_Q: 15.4 (18.0) |  |
| **P2\_W**  4 in 199 sites   2.0 %    effects > 10 perc. pnts.  (vice-versa in brackets)  P3\_H: 22.0 (14.7)   P1prime\_F: 21.0 (10.5) |  |
